# Cortical areas needed for choosing actions based on desires

**DOI:** 10.1093/brain/awx119

**Published:** 2017-05-24

**Authors:** Sanjay G. Manohar, Thomas Akam

**Affiliations:** 1 Nuffield Department of Clinical Neuroscience, University of Oxford, Oxford, UK; 2 Department of Experimental Psychology, University of Oxford; Fundação Champalimaud, Lisbon, Portugal

## Abstract

**This scientific commentary refers to ‘Selective impairment of goal-directed decision-making following lesions to the human ventromedial prefrontal cortex’, by Reber *et al.* 2017 (doi:10.1093/brain/awx105).**

**This scientific commentary refers to ‘Selective impairment of goal-directed decision-making following lesions to the human ventromedial prefrontal cortex’, by Reber *et al.* 2017 (doi:10.1093/brain/awx105).**

Lesions to prefrontal cortex lead to disinhibition, altered preferences and behavioural inflexibility. These clinical problems can be viewed as deficits in goal-directed behaviour. Goals enable coordinated planning of actions, and crucially, they can change depending on circumstances, permitting flexible behaviour. In this issue of *Brain*, Reber and co-workers (2017) demonstrate that patients with damage to ventromedial prefrontal cortex (vmPFC) show a specific deficit in flexibly adjusting behaviour to reflect a change in goal.

The study examines six patients with vmPFC lesions, and compares them to a control group with temporal lobe lesions and to a healthy group. The authors adapted a well-studied behavioural paradigm from the animal learning literature, termed outcome devaluation. Devaluation studies aim to test what a subject learns when they learn to perform an action to obtain a reward. Do they simply learn that the action is valuable, or do they learn that it leads to a specific outcome, the subjective value of which might vary depending on current goals? In the devaluation paradigm, the subject learns to perform two different actions to obtain two different rewarding outcomes, typically different foods. One of them is then devalued, for example by satiation or pairing with illness, so that it becomes subjectively less desirable. Finally, the tendency to perform both actions is assessed. If the subject has learned the specific outcomes that follow the actions, then devaluing one outcome should reduce the tendency to perform its corresponding action. If instead the subject has learned only whether an action is valuable, then subsequently devaluing its outcome will have no effect.

In Reber *et al.*’s study, participants learned to press two different keys to obtain two different foods, one sweet and the other savoury, delivered in capsules from two vending machines (Fig 1A). The foods were chosen to be initially equally desirable to the subject, and exposure to the two rewards was matched during learning. Then in a free-responding phase, participants pressed the buttons to obtain as much as they wanted of either food, to eat afterwards. One of the two food types was then devalued, by providing the subject with a bowlful and instructing them to eat as much as they could. Participants were then re-tested in a second free-responding phase, and were told that after the session, they would eat everything they obtained.

**Figure 1:**
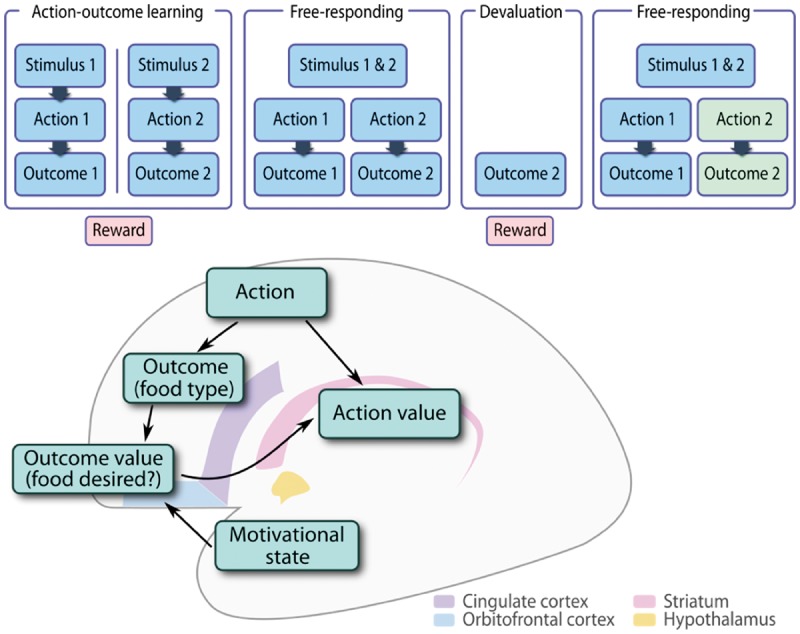
**Testing whether patients select actions by outcome desirability**. **(A)** Structure of the experiment. Participants initially learned that repeatedly pressing one button (Action 1) would lead to Outcome 1 (capsules of savoury food), and that another button (Action 2) would lead to Outcome 2 (capsules of sweet food). These foods were consumed during the learning phase. Then in a free-responding session, both actions could be performed to obtain as much of each food as desired, to be consumed afterwards. During the devaluation phase, participants were instructed to eat as much as possible of one outcome type. Finally, a second free-responding phase measured changes in the propensity to choose each action. If actions are chosen according to the desirability of the outcomes, then the action leading to the devalued outcome should be performed less often. (**B**) Possible schematic of computations for calculating action value. Simple reinforcement of actions leads to a direct estimation of an action’s value, i.e. how often the action has led to reward. In parallel, the specific outcomes (food types) that have been paired with the action are considered, and their subjective desirability evaluated in the current motivational state. This provides a context-sensitive estimate of the value of performing the action.

Healthy participants and lesion controls were less likely to choose the action that led to the devalued food in the final session, compared to baseline. This indicates that reductions in subjective value were appropriately translated into action. However, patients with vmPFC lesions chose the action that led to the devalued food just as often after devaluation as at baseline. Therefore, these patients failed to appropriately inhibit actions to obtain the devalued food. Participants also gave desirability ratings for each food before and after the devaluation. Following the devaluation, both patients and controls reported the food they were satiated on to be less desirable, suggesting that vmPFC lesions did not impair subjective re-evaluation. Therefore, despite appropriately reporting that they desired the food less, patients with vmPFC lesions did not use that information to choose the corresponding action less often. A very similar deficit is observed after lesions to monkey orbitofrontal cortex (West *et al.*, 2011; Rhodes and Murray, 2013).

The result adds a new twist to evidence from functional imaging in humans and physiological recordings in primates. In a range of studies, orbitofrontal activity correlates with the current subjective value of an outcome, whereas nearby regions of cingulate cortex are active when learning the outcome of an action (Levy and Glimcher, 2012; Bissonette and Roesch, 2016). This suggests a model in which goals are evaluated in ventral regions of the medial wall, to permit the selection of actions to obtain those goals in more dorsal areas (Kolling *et al.*, 2016). Goals and contingency must thus be coupled together to select actions appropriate to the current desire (Fig. 1B). The vmPFC lies at the intersection of these valuation and action selection regions. Previous lesion studies have suggested that vmPFC damage produces changes in preference, value comparison, future thinking and motivational modulation of action (Fellows, 2005; Wheeler and Fellows, 2008). But until now, the role of the vmPFC in flexible goal-directed action has not been tested directly in humans, because isolated lesions to this area are rare.

The design of the new study differs from classical outcome devaluation in one important respect; the vending machines still delivered outcomes (food capsules) during the post-devaluation test, though these were not consumed until later. In classical outcome devaluation, the post-devaluation test is performed in extinction with no further reward delivery (Dickinson and Balleine, 1994). The rationale for this is to ensure that any devaluation effect is solely due to the previously learned association between the action and its specific outcome. If outcomes are delivered during the test, new learning could reduce selection of the action that leads to the now devalued outcome. Specifically, a subject who had learned only that the action was valuable, but not the specific outcome that it led to, could still learn to respond less upon receiving outcomes known to be valueless after performing actions thought to be valuable. Thus, the test used here is in principle solvable without using action-outcome predictions. It remains possible that the healthy participants learned updated action values online to some extent during the devaluation test. The observed failure of the vmPFC patients to exhibit a devaluation effect could be consistent with a deficit of behavioural flexibility, rather than goal-directed behaviour specifically. This would fit with previous findings that vmPFC lesions impair deterministic and probabilistic reversal learning, without impairing acquisition (Fellows and Farah, 2003; Hornak *et al.*, 2004).

One key advance in this study is the very simple but effective experimental set-up. A dispenser allowed real physical foods to be delivered at the time of action. The simplicity of this design ensures that patients can understand the task in a direct way, unlike experiments involving more complex stimulus-response mappings with monetary rewards. It would be straightforward to adapt the experiment design such that the devaluation test was performed in extinction, by telling participants that the rewards earned will be delivered after the test period. With this change, the task would closely match that used in the animal literature to identify dissociations between brain regions involved in goal-directed and habitual behaviours. It would also be useful to contrast the current design with a control experiment in which, rather than devaluing the outcome, one of the actions simply stopped delivering rewards altogether. This would help isolate whether the deficit found in the current study is due to generally reduced behavioural flexibility or is specific to adjusting behaviour to reflect changes in outcome values due to shifts in motivational state.

The study is remarkable for bringing together the clinical intuition that frontal patients are unable to flexibly control and plan a course of action, with a rich corpus of non-human work on goal-directed behaviour. It is the first study to demonstrate that clinical difficulties in day-to-day planning may relate to the specific mechanism that links subjective value to action selection. Further refinements of the design would clarify some of the questions raised by this study. Goal-directed behaviour is likely to involve a range of brain areas, including dorsomedial and frontopolar cortex, but also subcortical nuclei such as the amygdala, ventral striatum, and hypothalamus (Dayan and Berridge, 2014). While this study confirms involvement of vmPFC, we cannot draw conclusions about the roles of other regions. It is also possible that the white matter pathways of the medial forebrain are critical for the devaluation effect. To answer those questions, further studies with larger cohorts involving lesions from a wider array of brain regions are needed. This would enable us to establish how action-outcome knowledge is combined with goal value and motivational drives, to produce true goal-directed behaviour.

Glossary

**Devaluation**: A behavioural paradigm that tests whether action-outcome associations are used to select actions, by reducing the value of an outcome—for example by satiation or pairing with illness.

**Goal-directed behaviour**: Our ability to choose an appropriate sequence of actions in order to achieve something we have in mind.

**Ventromedial prefrontal cortex (vmPFC)**: A loosely-defined area within prefrontal cortex including Brodmann areas 25, 14 and 32. It consists of the medial part of the orbital surface, and the ventral region of the medial wall of the frontal lobe.
